# Memory-Replay Knowledge Distillation

**DOI:** 10.3390/s21082792

**Published:** 2021-04-15

**Authors:** Jiyue Wang, Pei Zhang, Yanxiong Li

**Affiliations:** 1 School of Electronic and Information Engineering, South China University of Technology, Guangzhou 510641, China; eeyxli@scut.edu.cn; 2 School of Computer Science, Northwestern Polytechnical University, Xi’an 710072, China; cszhangpei@mail.nwpu.edu.cn

**Keywords:** Deep Neural Network, self-knowledge distillation, training trajectory, Knowledge Adjustment, Fully Connected Network, image classification, audio classification

## Abstract

Knowledge Distillation (KD), which transfers the knowledge from a teacher to a student network by penalizing their Kullback–Leibler (KL) divergence, is a widely used tool for Deep Neural Network (DNN) compression in intelligent sensor systems. Traditional KD uses pre-trained teacher, while self-KD distills its own knowledge to achieve better performance. The role of the teacher in self-KD is usually played by multi-branch peers or the identical sample with different augmentation. However, the mentioned self-KD methods above have their limitation for widespread use. The former needs to redesign the DNN for different tasks, and the latter relies on the effectiveness of the augmentation method. To avoid the limitation above, we propose a new self-KD method, Memory-replay Knowledge Distillation (MrKD), that uses the historical models as teachers. Firstly, we propose a novel self-KD training method that penalizes the KD loss between the current model’s output distributions and its backup outputs on the training trajectory. This strategy can regularize the model with its historical output distribution space to stabilize the learning. Secondly, a simple Fully Connected Network (FCN) is applied to ensemble the historical teacher’s output for a better guidance. Finally, to ensure the teacher outputs offer the right class as ground truth, we correct the teacher logit output by the Knowledge Adjustment (KA) method. Experiments on the image (dataset CIFAR-100, CIFAR-10, and CINIC-10) and audio (dataset DCASE) classification tasks show that MrKD improves single model training and working efficiently across different datasets. In contrast to the existing fancy self-KD methods with various external knowledge, the effectiveness of MrKD sheds light on the usually abandoned historical models during the training trajectory.

## 1. Introduction

Despite the appealing performance of the Deep Neural Networks (DNNs), as their parameter size grows dramatically and consumes enormous computational resources [1,2,3], there is a trend to use light but powerful models [4,5,6,7] to match the low-performance sensor devices. With a carefully designed supernet space and model searching strategy, Neural Architecture Search(NAS) techniques [8,9] can find proper models to fit various hardware and sensor requirements (flops, memory).

Knowledge Distillation (KD) [10] is also a popular technique that has been investigated quite intensively for model compression recently. KD compressed the knowledge from the teacher model, which is a larger model or a set of multiple models, to a single small student model. The teacher model is trained stand-alone beforehand. In the procedure of student training, the teacher model’s parameters are frozen, and the Kullback–Leibler (KL) divergence loss between their output probabilities is penalized. KD is applied to various tasks. Cho et al. [11] used group sparsity regularization to improve student learning. Park et al. [12] introduced channel and spatial correlation loss and the adaptive Cross-Entropy (CE) loss for applying KD to semantic segmentation problem. Choi et al. [13] investigated KD on serial number recognition task and applied the Bayesian optimization method to automatically tune KD’s hyper-parameters. Chechlinski et al. [14] develop a light system for weeds and crops identification with KD.

Besides model compression, KD can also improve network training, such as multiple models collaborative learning [15,16,17], and single model self-KD [18,19,20,21]. Zhang et al. [18] proposed a self-KD method that divides a single network into several sections and the knowledge in the deepest classifier of the network is squeezed into the shallower ones. Xu et al. [20] designed a more elegant self-KD mechanism to transfer knowledge between different augmented versions of identical training data. As an online training method, Self-KD is more suitable for applications on intelligent sensors with limited memory and computational resources than the two-stage traditional KD method, which needs to train a cumbersome teacher beforehand. However, the mentioned self-KD methods above have their limitation for widespread use. Multi-branch KD methods [18,21] need to redesign the DNN for different tasks, and the data-distortion methods [20,22] rely on the effectiveness of the augmentation method.

**Figure 1:**
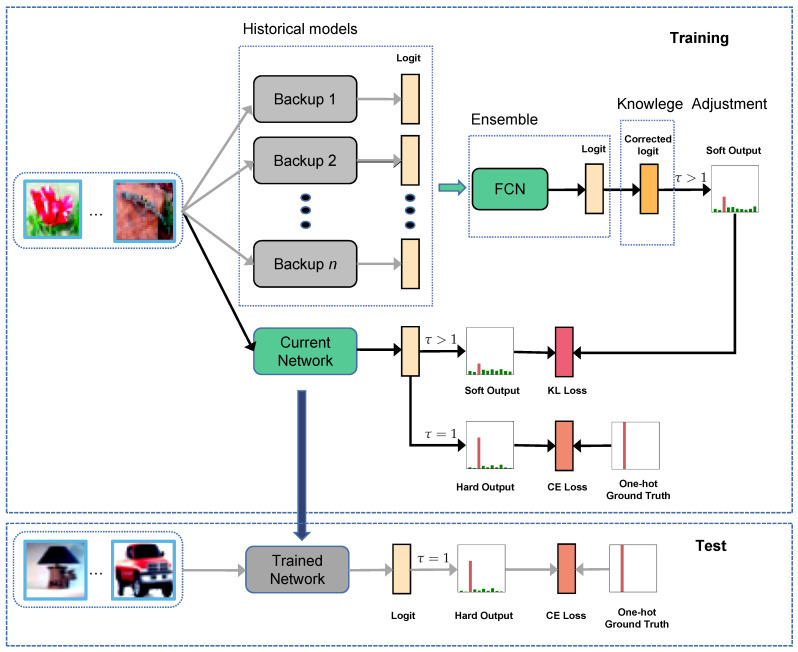
The framework of our proposed memory replay Knowledge Method with Fully Connected Network and Knowledge Adjustment.

The motivation of this article is to try to provide a universal KD approach that can be combined with different DNNs and applied to different tasks with no extra modification. The proposed self-KD method is called Memory replay Knowledge Distillation (MrKD), illustrated in Figure 1 above. No extra model [15,16] or structure [18] is required in our strategy: the knowledge is distilled from the model backups during the training trajectory. The method is based on the assumption that a student can be improved by reflecting on his own experience. In the training session, *n* model backups are used as teachers. In every κ steps during the training procedure, the network backup *n*’ parameter ${\hat{\theta }}_{n}$ is updated as backup *n* − 1, while ${\hat{\theta }}_{n-1}$ is updated as ${\hat{\theta }}_{n-2}$, and so on. Finally, ${\hat{\theta }}_{1}$ is updated as the current model parameters θ. Besides the traditional supervised learning CE loss, the averaged KL loss between the current and the backup models will also be penalized for regularizing the model to a more general solution. In the test session, since the label information is unknown, the auxiliary KL loss with the corrected logits is abandoned. The network predicts test data similar to a regularly trained network.

This model backup strategy is rarely used in conventional supervised learning but is a common practice in the Deep Reinforcement Learning (DRL) method [23] for the target network renewal. By offering a stable temporal q-value for the current network training, the target network design is critical to DRL for achieving a robust network and surpassing human-level control on Atari games. Similarly, in the supervised learning problem, the whole stochastic gradient descent (SGD) training procedure can be regarded as a Markov Chain trajectory sampled from a dynamic transition distribution parameterized by learning rate, mini-batch size, sampling order, and the model weight initialization [24]. The diverse distribution of the model backup κ steps ago can be an informational reference for the model to achieve a more general minimum.

Although it is convenient to use the training trajectory model backups as teachers, these teachers have obvious flaws comparing to multiple model methods [15,16]: inaccuracy and similarity. As we mentioned above, since the teachers are historical model backups that are inevitably worse than the current student, the plain MrKD method may degrade the student learning. Furthermore, because the teachers and the student belong to the same training procedure, their similarity would also deteriorate the performance.

To relieve the disadvantages of MrKD above, we ensemble the backup teachers by a simple Fully Connected Network (FCN). As Figure 1 shows, by ensembling the backup output ligits, the FCN acts as the new teacher for the current student network training. The student is trained with the CE loss and KL loss as MrKD, whereas the FCN is only trained by the KL loss. The ensembling output is usually more accurate than the backup outputs. Meanwhile, by re-processing the backup logits, FCN can decouple the similarity between backups and the current student. In conclusion, MrKD-FCN can alleviate the inaccuracy and similarity problems of MrKD. Additionally, we introduce the Knowledge Adjustment (KA) [25] method to assure the maximum of each teacher logit is reached at ground truth label by swapping the value of the ground truth with the value of the predicted class.

The contributions of this work are summarized as follows:

1. We propose a new self-distillation method, MrKD, which distills knowledge from the training trajectory model backups. By FCN ensembling and Knowledge Adjustment, MrKD offers reliable knowledge to improve the generalization of current training network.
2. The Knowledge Adjustment method was originally used in static teacher-student KD methods. For the first time, we apply KA in self-KD to reduce the misleading risk of the imperfect teacher.
3. The proposed methods are evaluated exhaustively on image classification datasets (CIFAR-100, CIFAR-10 [26], and CINIC-10 [27]) with various networks (ResNet [1], WideResNet [28], ResNeXt [29]). We also conduct MrKD on audio datasets DCASE’18 acoustic scene classification (ASC) [30] and DCASE’20 Low Complexity ASC [31]. Our experiments demonstrate that MrKD can help improve model training across different network architectures and different datasets consistently.

The remainder of the paper is organized as follows. In Section 2, we briefly review the related work, and Section 3 describes the MrKD method. Section 4 shows the experimental results, and Section 6 summarizes the whole work.

## 2. Related Work

Self-Knowledge Distillation: Furlanello et al. [15] proposed a Born Again Network (BAN) that a teacher parameterized identically to the student can improve student training significantly. For the next iteration of training, the trained student is set as the teacher. However, the recurrent distillation of BAN requires high computation and storage costs. Zhang et al. [18] divided a single network into several branches connected with extra bottlenecks and densely connected layers to comprise multiple classifiers. Then the deepest classifier acts as a teacher to guide the shallower branches learning by KL loss. Their study of self-KD is promising; they claimed that the teacher branch improves the shallower sections’ learning features. Ref. [21] deepened the shallower section’s bottleneck classifier and applied mutual learning distillation instead of the teacher-student method and achieve better performance. This improvement of MSD indicates that the self-distillation method can be regarded as a Deep Mutual Learning (DML) [16] method of four peers with different low-level weight sharing. We evaluate four-model DML directly and found comparable results. Except with fewer parameters, this self-distillation method [18] can also be regarded as a multi-model KD method as DML. These network remodeling or model ensembling methods [18,32,33] have the limitation of generalization and flexibility.

Furthermore, there are other types of self-KD methods that do not need extra model peers; we roughly categorize them as data-based and model backup-based self-KD. Data-based self-KD tried to regularize the model output consistency of similar training samples, such as augmented data and original data [22], or samples from the same classes (CS-KD [19]). However, the former method relies on the augmentation method’s efficacy, and the latter needs a carefully designed training procedure. The model backup-based self-KD is introduced in the next paragraph.

KD with historical models: Ref. [34] revealed that larger models are not making good teachers because of capacity mismatching: small students cannot mimic large teachers. To alleviate the capacity mismatching problem, Ref. [35] introduces multi-step KD, which uses an intermediate-sized model (teacher assistant) to bridge the gap between the student and teacher. Route Constrained Optimization (RCO) [36] supervises the student model with some anchor points selected from the route in parameter space that the teacher pass by, instead of the converged teacher model. Our MrKD method extracts the anchor points progressively from the student itself during the training. Inspired by the fact that averaging model weights over training steps tend to find a flatter solution [37], the Mean Teacher [38] method distilled the knowledge from a teacher that averages successive steps model weights and improved the performance of semi-supervised tasks. Ref. [39] fine-tuned the BERT model in Natural Language Processing problems by distilling the knowledge of the averaged weight parameter of κ recent steps. The recent time steps historical model KD can help semi-supervised learning or model fine-tuning but scarcely improve common classification problems.

**Figure 2:**
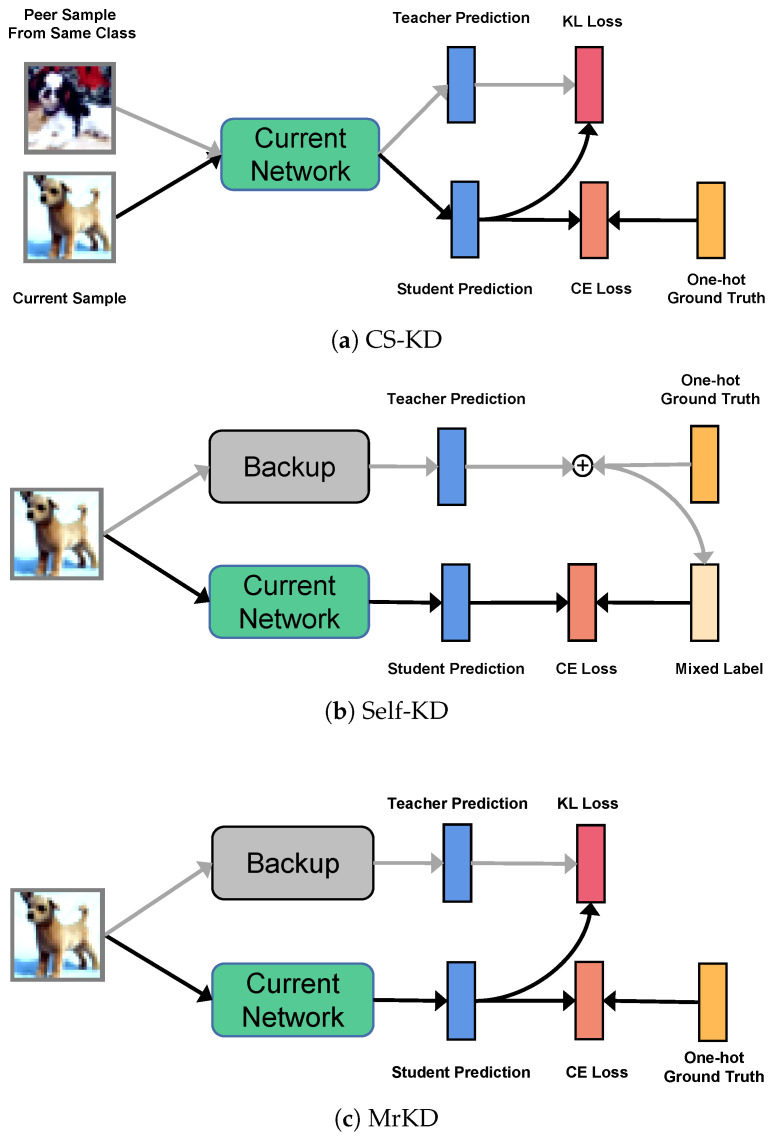
Simplified graphical illustration for different self-knowledge distillation methods. (**a**) Class-wise self-Knowledge Distillation [19]; (**b**) self-Knowledge Distillation [21]; (**c**) memory replay Knowledge Distillation.

As Figure 2b shows, Kim et al. [40] proposed Self-KD, which progressively distills a model’s own knowledge one epoch ago to soften hard targets during training. Their work also used the historical model, except that they use the historical model output to smooth the one-hot ground truth instead of being a standalone teacher. Self-KD fixed the update frequency κ to one epoch, while MrKD reveals that the model backups far away from the current training can regularize supervised learning effectively. A properly tuned κ can effectively improve the performance of MrKD.

Summary: Some of the typical KD methods mentioned above are summarized in Table 1 with their implementation and computation complexity. MrKD has advantages in every aspect. Firstly, as an online KD method, MrKD does not need to pre-train a teacher. Secondly, since MrKD uses its own historical models, the parameters engaged do not need to be doubled as other KD methods rely on peer output. Thirdly, MrKD only adjusts some training procedures; the model structure is untouched. Finally, compared to the collaborative KD method (DML and MSD), MrKD is a single model knowledge method learning from its own backups; thus, MrKD only needs one backward propagation in one training step. Overall, as a self-KD method, MrKD has many advantages for application and is worthy of further study.

## 3. Method

In this section, we first formulate the traditional knowledge distillation method (Section 3.1). Next, we introduce the plain self-knowledge distillation method using historical models as teachers in Section 3.2. Then, we improve it by reprocessing the model output logits with a fully connected network (Section 3.3) and knowledge adjustment (Section 3.4). Finally, we summarize our proposed full MrKD method in Section 3.5.

### 3.1. Knowledge Distillation

We consider a standard classification problem. Given a training dataset D = {(x${}^{i}$, y${}^{i}$)}${}_{i=1}^{N}$, where *x${}^{i}$* is the *i*${}_{th}$ sample from M classes and *y*${}^{i}$ ∈ {1, 2, …, M} is the corresponding label of sample *x*${}^{i}$, the parameters θ of a deep neural network (DNN) that best fit to the dataset need to be determined.

The softmax function is employed to calculate the *m*${}_{th}$ class probability from a given model:

(1) $$p_{m}\left(\theta ;\tau \right)=\frac{exp(z_{m}\left(\theta \right)/\tau )}{\sum_{i=1}^{M}exp(z_{i}\left(\theta \right)/\tau )}.$$

Here *z*${}_{m}\left(\theta \right)$ is the *m${}_{th}$* logit output of the model’s fully connected layer. τ indicates the temperature of softmax distribution normally set to 1 in traditional cross-entropy loss but greater than 1 in knowledge distillation loss [10]. A larger τ means a softer probability distribution that reveals more detail than a hard softmax output (τ = 1).

Firstly, for M-class classification, the traditional cross-entropy loss of a sample is as follows:

(2) $$L_{CE}\left(p\left(\theta ;\tau =1\right),q\right)=-\sum_{m=1}^{M}q_{m}log\left(p_{m}\left(\theta ;\tau =1\right)\right),$$

where *q*${}_{m}$ is the *m*${}_{th}$ element of one-hot label vector *q*. Note that the temperature τ is set to 1.

In the KD method, a teacher network is trained beforehand. The parameter of the pre-trained teacher is then frozen, and only forward-propagation is conducted during the student training. The teacher outputs a corresponding logit $z^{t}$. To transfer the knowledge from the teacher model to the student, KL Divergence between their output probabilities is penalized:

(3) $$L_{KL}(p\left(\theta^{t};\tau \right)||p\left(\theta ;\tau \right))=\sum_{m=1}^{M}p_{m}\left(\theta^{t};\tau \right)log\left(\frac{p_{m}\left(\theta^{t};\tau \right)}{p_{m}\left(\theta ;\tau \right)}\right),$$

Here the temperature τ is a hyper-parameter need to be tuned, and the $p_{m}\left(\theta^{t};\tau \right)$ is obtained by Equation (1) with $z_{m}\left(\theta^{t}\right)$. During training, the KD method calculates the sum of two losses above with a hyper-parameter α:

(4) $$L_{KD}=\left(1-\alpha \right)\times L_{CE}\left(p\left(\theta ;\tau =1\right),q\right)+\alpha \times \tau^{2}\times L_{KL}(p\left(\theta^{t};\tau \right)||p\left(\theta ;\tau \right)),$$

where $\tau^{2}$ is a factor in ensuring that the relative contribution of the ground-truth label and teacher output distribution remains roughly unchanged [10].

### 3.2. Knowledge Distillation with Historical Models

**Figure 3:**
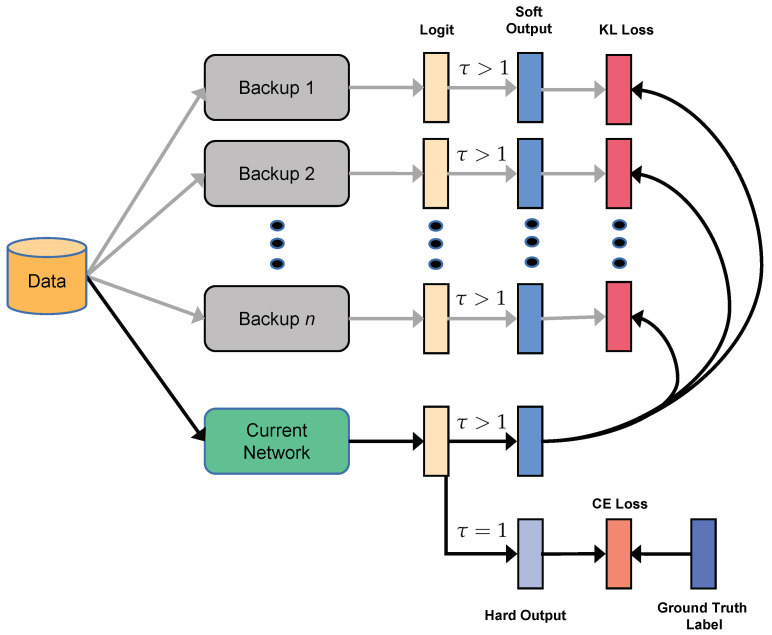
The framework of MrKD-plain without Fully Connected Network (FCN) ensemble and Knowledge Adjustment (KA).

Unlike the traditional KD method above, the historical models during the training trajectory can also help the current model training. Figure 3 shows the plain self-knowledge distillation method without using the other two components. In every κ steps during the training, the backup model weights $\hat{\theta }$ will be updated to the current model θ. The identical structure model with parameter $\hat{\theta }$ is used as a teacher in Equation (4). Thus, the plain MrKD loss is:

(5) $$L_{MrKD-plain}=\left(1-\alpha \right)\times L_{CE}\left(p\left(\theta ;\tau =1\right),q\right)+\alpha \times \tau^{2}\times L_{KL}(p\left(\hat{\theta };\tau \right)||p\left(\theta ;\tau \right)).$$

The proposed method can extend to *n* memory copies ${\hat{\theta }}^{1}$, …, ${\hat{\theta }}^{n}$, with κ training steps interval. The KL loss in Equation (5) is substituted as:

(6) $$L_{KL}(p\left({\hat{\theta }}^{1};\tau \right),\ldots ,p\left({\hat{\theta }}^{n};\tau \right)||p\left(\theta ;\tau \right))=\frac{1}{n}\sum_{i=1}^{n}L_{KL}(p\left({\hat{\theta }}^{i};\tau \right)||p\left(\theta ;\tau \right)).$$

The training procedure is shown in Algorithm 1. With every κ steps, all the model copies’ parameters ${\hat{\theta }}^{1}$, … ${\hat{\theta }}^{n}$, are updated recursively. In each step, a mini-batch *d* is sampled and fed into the current model and its copies. With the models’ logit outputs *z*(θ), *z*(${\hat{\theta }}^{1}$), …, *z*(${\hat{\theta }}^{n}$), we can get the probabilities of mini-batch *d* by Equation (1). Then the loss is calculated by Equation (5). Finally, the current model parameters θ are updated by SGD as Equation (7). Note that this algorithm can benefit from multiple GPU training. If n+1 GPUs are available, where n is the number of copies, distributed forward pass can be implemented for n+1 models, then the training time will be identical to the standard training method.
**Algorithm 1** Self-Knowledge Distillation with Historical Models**Require:** training set D, learning rate $\lambda_{t}$, kd loss ratio α, copy step interval κ, copy amount *n*, temperature τ, total training steps T**Initilize:** model parameters θ, ${\hat{\theta }}^{1}$, …, ${\hat{\theta }}^{n}$**for***t* = 1, …, T **do** **if** (tmodκ) == 0 **then**  **for** *i* = *n*, …, 2 **do**   ${\hat{\theta }}^{i}$ := ${\hat{\theta }}^{i-1}$  **end for**  ${\hat{\theta }}^{1}$ := θ **end if** Sample a mini-batch of data *d* form D Feed *d* to networks θ, ${\hat{\theta }}^{1}$, …, ${\hat{\theta }}^{n}$, and get the logits *z*(θ), *z*(${\hat{\theta }}^{1}$), …, *z*(${\hat{\theta }}^{n}$) Compute the predictions *p*(θ; τ = 1), *p*(θ; τ), *p*(${\hat{\theta }}^{1}$; τ), …, *p*(${\hat{\theta }}^{n}$; τ) by Equation (1) Compute loss $L_{MrKD-plain}$ (θ) by Equation (5) Update θ with SGD
(7)$\theta :=\theta -\lambda_{t}\frac{\partial L_{MrKD-plain}}{\partial \theta }$**end for****Output:**θ

### 3.3. Fully Connected Network Ensemble

In MrKD-plain, the historical models, which are inevitably worse than the current student, have the risk of degrading the student learning. Moreover, because the teachers and the student belong to the same training procedure, the similarity between them would also deteriorate the performance.

**Figure 4:**
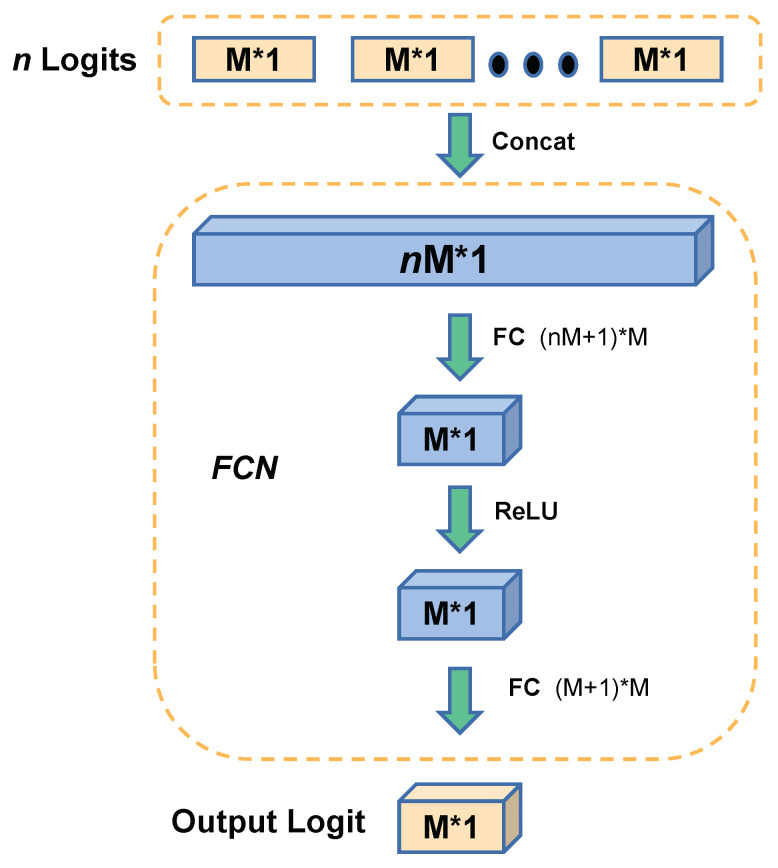
The framework of the Fully Connected Network.

Overall, to deal with the inaccuracy and similarity issue of plain MrKD, we introduce the FCN ensemble. Instead of adopting the training trajectory models’ outputs as teachers directly in the previous section, the backup output logits ${\hat{z}}_{1}$, ${\hat{z}}_{2}$, … ${\hat{z}}_{n}$ with size *M* * 1 are concatenated as a single *nM* * 1 feature vector and fed into a two-layers FCN. As Figure 4 shows, the FCN can further process the information that backups offered and output the ensembled logit ${\hat{z}}_{ens}$.

The parameter sizes of the two layers including the bias are (*nM* + 1) × *M*, (*M* + 1) × *M* respectively, where *M* is the number of classes. Thus, the total parameter number is (*nM* + 1) × *M* + (*M* + 1) × *M*. For example, on the CIFAR-100 dataset which the class number *M* is 100, and with *n* set to 3, the parameter size of FCN is 420. This small parameter number is negligible comparing to the main deep neural networks, which always have millions or billions of parameters. Thus, the FCN procedure consumes negligible computational resources than the plain MrKD method.

### 3.4. Knowledge Adjustment

**Figure 5:**
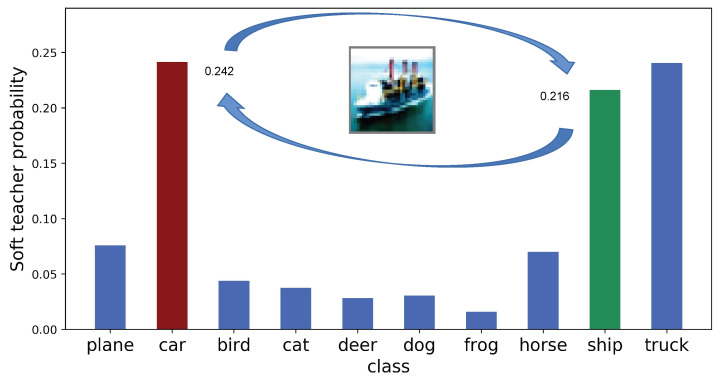
Knowledge Adjustment of a wrong probability offered by an imperfect teacher. The distribution is from a sample of the CIFAR-10 training dataset, whose ground truth label is ‘ship’, but the teacher’s prediction is ‘car’. Their values are exchanged.

To further improve the teacher probability distribution, we adopt the Knowledge Adjustment (KA) [25] method. Given the output $z_{ensemble}$(ϕ) of FCN, KA swaps the value of ground truth (the theoretical maximum) with the value of predicted class (the predicted maximum) and obtains the corrected logit $z_{correct}\left(\phi \right)$. As shown in Figure 5 below, KA assures the maximum of each logit is reached at the ground truth label.

The KA method was originally used in static teacher-student KD methods for model compression, where the teacher is pre-trained. Since the teacher always has a larger capacity and is well trained before teaching the student learning. The teacher tends to overfit the training set and rarely makes mistakes on it. Thus, KA helps marginally on the traditional KD training. For online knowledge distillation like our method, where the teacher accuracy is growing up as the student in the training procedure, the miss classification issue is more serious. In this case, KA is more critical to reduce the misleading risk of the imperfect teacher.

### 3.5. Memory Replay Knowledge Distillation

Over all, the total loss of MrKD with FCN and KA is as folows:

(8) $$L_{MrKD}=\left(1-\alpha \right)L_{CE}\left(p\left(\theta ;\tau =1\right),q\right)+\alpha \times \tau^{2}L_{KL}(p_{correct}\left(\phi ;\tau \right)||p\left(\theta ;\tau \right)),$$

note that $p_{correct}\left(\phi ;\tau \right)$ is the soft target of $z_{correct}\left(\phi \right)$ obtain by Equation (1). Additionally, the parameter ϕ for FCN is also trained by the second term of Equation (8).

The training procedure of our proposed MrKD with Fully Connected Network and Knowledge Adjustment is shown in Algorithm 2. The different part comparing to Algorithm 1 is highlighted in bold. With the backups’ logit outputs *z*(θ), *z*(${\hat{\theta }}^{1}$), …, *z*(${\hat{\theta }}^{n}$), we can get the ensembled logit $z_{ensemble}$(ϕ). Then the $z_{correct}\left(\phi \right)$ is obtained by Knowledge Adjustment refer to ground truth label *p*. Based on the logit values, we can get the probabilities *p*(θ; τ = 1), *p*(θ; τ), *p*${}_{ensemble}\left(\phi ;\tau \right)$ of mini-batch *d* by Equation (1) and the loss is calculated by Equation (8). Finally, the current model parameters θ and the FCN parameter ϕ are updated by SGD as Equations (9) and (10) respectively. Note that this algorithm can still benefit from multiple GPU training as mentioned in Section 3.2.
**Algorithm 2** Memory Replay Knowledge Distillation**Require:** training set D, learning rate $\lambda_{t}$, kd loss ratio α, copy step interval κ, copy amount *n*, temperature τ, total training steps T**Initilize: model parameters θ, ${\hat{\theta }}_{1}$, …, ${\hat{\theta }}_{n}$, ϕ****for***t* = 1, …, T **do** **if** (tmodκ) == 0 **then**  **for** *i* = *n*, …, 2 **do**   ${\hat{\theta }}_{i}$ := ${\hat{\theta }}_{i-1}$  **end for**  ${\hat{\theta }}_{1}$ := θ **end if** Sample a mini-batch of data *d* and label *p* form D Feed *d* to networks and get logits *z*(θ), *z*(${\hat{\theta }}^{1}$), …, *z*(${\hat{\theta }}^{n}$) **Feed *z* (${\hat{\theta }}^{1}$), …, *z* (${\hat{\theta }}^{n}$) to Fully Connected Network and get logits $z_{ensemble}$(ϕ)** **Correct the value $z_{ensemble}$(ϕ) to $z_{correct}$(ϕ) refer to label *p* by Knowledge Adjustment method** **Compute the predictions *p* (θ; τ = 1), *p* (θ; τ), *p*${}_{ensemble}$(ϕ;τ) by Equation (1)** Compute loss $L_{MrKD}$ (θ) by Equation (8) Update θ and ϕ with stochastic gradient descent:
(9)$$\theta :=\theta -\lambda_{t}\frac{\partial L_{MrKD}}{\partial \theta }$$
(10)$\phi :=\phi -\lambda_{t}\frac{\partial L_{MrKD}}{\partial \phi }$**end for****Output:**θ

## 4. Experiments

In this section, we conduct experiments to evaluate MrKD on five datasets for image and audio classification: CIFAR100, CIFAR10 [26], CINIC10 [27] DCASE’18 ASC [30] and DCASE’20 Low Complexity [31]. For a fair comparison, all results on the same dataset are obtained with the identical setting. We implement the networks and training procedures in PyTorch and conduct all experiments on a single NVIDIA TITAN RTX GPU. Besides baseline and MrKD, we also provide the results of two peer self-knowledge distillation methods, self-KD [40] and MSD [21], that are introduced in Section 2.

### 4.1. CIFAR-100

**Figure 6:**
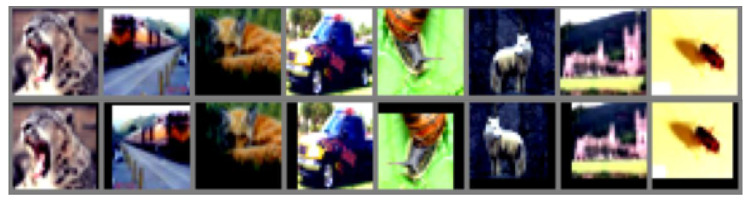
Samples from the CIRFAR-100 dataset. Upper: original image. Lower: augmented image. The labels from left to right: leopard, train, fox, truck, snail, wolf, castle, and cockroach.

The CIFAR-100 [26] dataset consisted of 50,000 training images and 10,000 test 32 × 32 color images in 100 classes, with 600 images per class in total. A random horizontal flip and crop with 4 pixels zero-padding weere carried out for data augmentation in the training procedure. The networks used below were implemented as their official papers for 32 × 32 images, including ResNet [1], WideResNet [28], ResNeXt [28]. See Figure 6.

For all runs, including the baselines, we trained a total epoch of 200, with batch size 128. The initial learning rate of 0.1 decreased to zero with linear annealing. The SGD optimizer was used with a weight decay of 0.0001, and momentum was set to 0.9. We averaged the last epoch results of four runs for all presented results because choosing the best epoch results was prone to benefiting unstable and oscillating configurations.

Experimental results are shown in Table 2. The best result for every network is in bold. It can be observed in Table 2 that MrKD improved the baseline consistently. With historical model teachers’ knowledge, MrKD decreased the error rate from 1.03% to 1.96% on the CIFAR100 test set. Although MSD obtained competitive results for some networks, we argue that MSD was a multi-branch method that needed to redesign each of the networks. On the other hand, self-KD, which smoothed the one hot label by a historical teacher, reduced the error rate not as significantly as MSD and MrKD.

Influence of each component: We empirically demonstrate the influence of MrKD with each component. Table 3 shows that the plain historical model distillation without FCN ensemble and Knowledge Adjustment could improve the networks from 0.6% to 1.2%. The FCN ensemble could decrease the error rate further by around 0.4–0.7%. Finally, compared to MrKD without KA, the full MrKD method showed more improvement when the networks went deeper. We argue that a deeper network was more sensitive to the correctness of the teacher distribution. Thus ResNet-164 and WRN-28-10 benefited more from KA than their shallower siblings.

Update frequency κ: The critical hyper-parameter for the MrKD method was the model backups’ update frequency κ. Following the setting in Section 3.1, we evaluated MrKD with different κ on the CIFAR-100 dataset within the range of {1/391, 4/391, 10/391, 40/391, 0.25, 1, 2.5, 10, 25, 50, 100, 200}. Note that the unit of κ was the epoch. As the batch size we set was 128 on CIFAR-100, the total iteration of an epoch was 391; thus, the κ = 10/391 meant we updated the copies every 10 steps, and κ = 200 means that we never updated the copies during the 200 epochs training. Overall, the range selected above covered the frequency from updating in each step to never renewing the copies during the whole training procedure. The widest range allowed us to observe the influence of update frequency κ thoroughly. The control variates method was used below to show the result, which meant that we set other hyper-parameters to the optimal value except for the one we wanted to evaluate.

**Figure 7:**
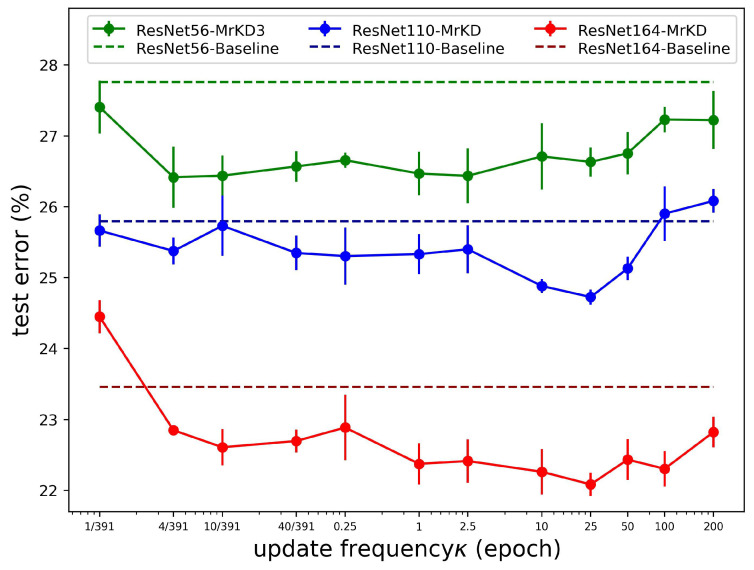
Performance on CIFAR-100 with different update frequency κ.

In Figure 7, we can see that if the step interval of the model backups was quite small, the error rate rose because the copy was too similar to the current model, then the regularization would not be helpful and may stumble the current model from learning. On the other hand, if the step was too large, the copies would be worse and lagging, then MrKD would also mislead and destabilize the learning. In conclusion, two ambivalent factors influenced the performance of MrKD while κ was changing: accuracy and diversity. For high accuracy, we needed to be updated the copies frequently, while for diversity, the copies needed to be far from the current model.

The shallower model (ResNet56) was relatively insensitive to κ. On ResNet110 and ResNet164, we can see clearly in Figure 7 that the optimum value of κ was 25. The short standard deviation bars indicated that the optimal values were very stable. These optimal values of κ were out of our expectation because updating copies every 25 epochs meant more than a 10% rise of training error than the current model. The large update step interval indicated that diversity was more important than accuracy.

**Figure 8:**
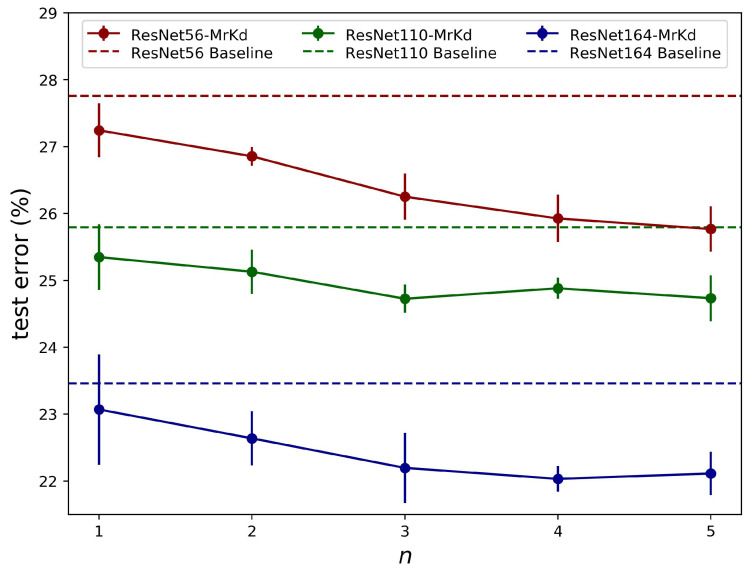
Performance on CIFAR-100 with different copy amount *n*.

Copy amount *n*: Figure 8 demonstrates the influence of more model backups being available. For ResNet-110 and ResNet-164, the performance gain was saturated with around three historical models. However, on shallower networks ResNet-56, more improvement was obtained with five copies. A similar trend could be found from multiple model distillation methods [15,16,17]. As our experiments showed in Section 4.1–Section 4.3, setting copies to three was a reasonable choice that could achieve significant improvement, yet, for shallower networks, more than three copies were worth trying for further improvement if computation resources were sufficient.

Fully Connected Network: For the Fully Connected Network, we only used the KL loss to update the parameters. Table 4 shows the loss options. Compared to the baseline, the plain CE loss improved the least. The KL loss between the FCN output and the model output decreased the error rate by 0.3% to 0.5%. The combination of CE and KL loss obtained similar results as KL loss only. We used the KL loss for ensembling only as of the online KD methods [41,42].

**Figure 9:**
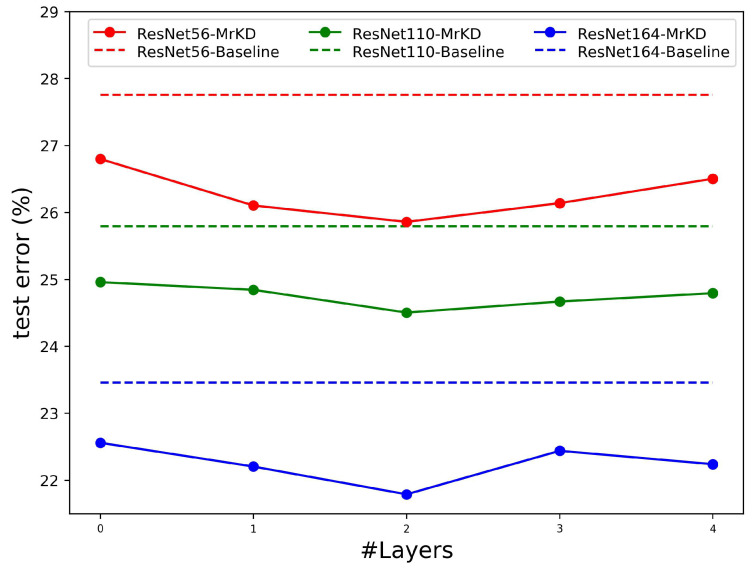
Performance of MrKD on CIFAR-100 with different depth FCN.

To determine the depth of FCN, we evaluated different layers for CIFAR-100 classification. Note that layers = 0 indicates the logits were averaged evenly with no parameter in FCN. As illustrated in Figure 9, MrKD achieved the optimal improvement when layer number equals two. With fewer layers, the FCN was be too simple to conduct the model copy logits. With the network going too deep, the FCN was prone to overfit the training set and impair the performance of MrKD.

### 4.2. CIFAR-10 and CINIC-10

**Figure 10:**
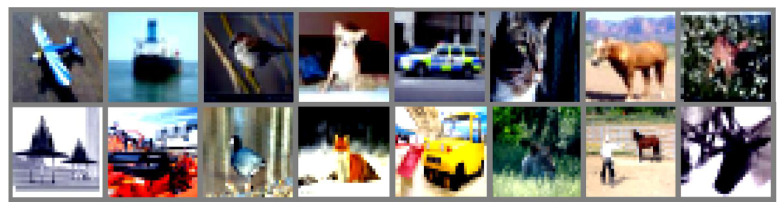
Samples from the CINIC-10 dataset. **Upper**: inherit from the CIFAR-10 dataset. **Lower**: extended from ImageNet dataset. The labels from left to right: plane, ship, bird, dog, car, cat, horse, and deer.

In CIFAR-10 [26] dataset experiments, the official divide of training data and test data was used, consisting of 50,000 images and 10,000 images, respectively, with a resolution of 32 × 32. As Figure 10 shows, the CINIC-10 [27] dataset was an extended version of CIFAR-10. It contained all images from the CIFAR-10 dataset and derived 210,000 images downsampled to 32 × 32 from the ImageNet dataset. Like CIFAR-100 implementation, a random horizontal flip and crop with four pixels zero-padding was for the training set. For CIFAR-10 and CINIC-10, we used the same hyper-parameters as on CIFAR-100 to keep universality. We believed that there would be better results on both datasets through a thorough search than we report in this paper.

Table 5 and Table 6 show similar improvements as in Table 2. MrKD improved the network error rates of CINIC-10 from 0.70 % to 1.24% and improved CIFAR-10 from 0.50% to 0.61%. Compared to the performance on CIFAR-100, it should be noted that self-KD performs better on 10-class classification datasets, which were more manageable and had less error rate gap between the training set and test set. Especially on the CIFAR-10 dataset, self-KD outperformed our method on WRN-16-8 and ResNeXt-29 network. We argue that the networks distilled less information on 10-class datasets problems. Furthermore, the gap between the test and training error rate on CIFAR10 was much lower than on CIFAR100; then, KD methods’ generalization effect was not significant. In this case, the smoothed target of self-KD may have become more effective than KD loss in MSD and MrKD.

### 4.3. DCASE Datasets

Acoustic scene classification (ASC) is a regular task in the Detection and Classification of Acoustic Scene and Event (DCASE) challenge. The objective of ASC is to categorize the short audio samples into predefined acoustic scene classes using the supervised learning method. In this section, the proposed MrKD is evaluated on two ASC datasets. The results presented are obtained by the official development dataset train/test split in which 70% of the data for each class is included for training, 30% for testing.

**Figure 11:**
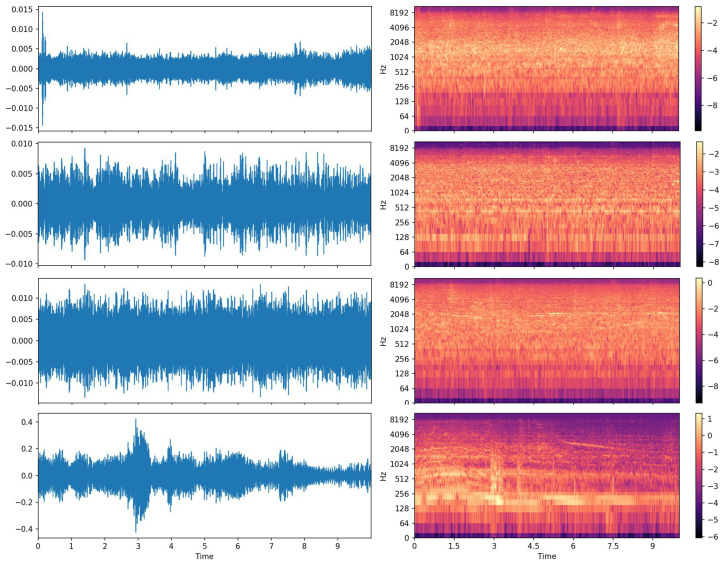
The 10-s audio clips from DCASE’18 ASC [30]. **Left**: the raw waveform data. **Right**: the corresponding log Mel spectrogram. The acoustic scenes from top to down: airport, park, shopping mall, bus.

DCASE’18 ASC [30]: The dataset contained 8640 audio segments of 10-s length, where 6122 clips were included in the training set and 2518 clips in the test subset. The 10 acoustic scenes were airport, shopping mall, metro station, pedestrian street, public square, street, traveling by tram, bus and underground, and urban park. As Figure 11 shows, the 10-s audio clips were down-sampled to 22.05 kHz. For feature extraction, the perceptually weighted Mel spectrograms were computed similar to Koutini et al. [43]. The result was a 256 × 431 tensor with 256 Mel frequency bins and 431 frames.

**Figure 12:**
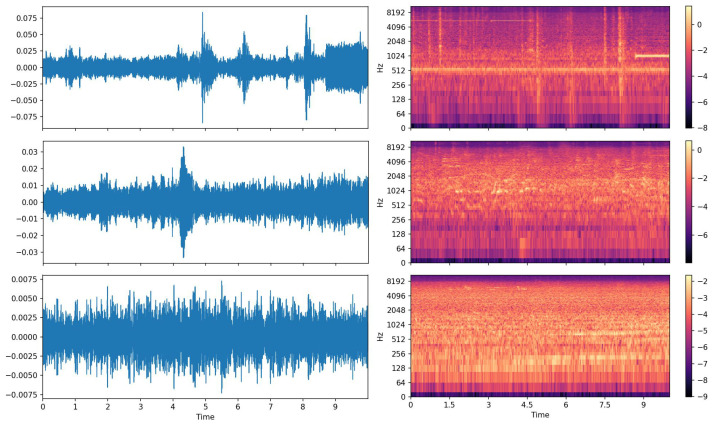
The 10-s audio clips from DCASE’20 Low Complexity ASC [31]. **Left**: the raw waveform data. **Right**: the corresponding log Mel spectrogram. The acoustic scenes from top to down: transportation (traveling by tram), indoor (metro station), outdoor (park).

DCASE’20 Low Complexity ASC [31] was a three-class supervised learning dataset that comprised 14,400 segments of 10-s length. The data were recorded from 10 acoustic scenes as with DCASE’18 ASC and summarized into three categories, indoor, outdoor, and transportation. As Figure 12 shows, the feature extraction procedure was the same as the DCASE’18 ASC experiment. We chose this dataset because it was a low complexity three-class classification task and required the model parameter less than the 500 KB size limit.

For both datasets, the presented baselines are the corresponding model trained with only regular classification cross-entropy loss. For DCASE’18 ASC, the network CP-ResNet from Koutini et al. [44] was used as the baseline to evaluate the methods proposed in this paper. We ran a total epoch of 200, with batch size 10. The learning rate was set to 0.0001 and decreased to zero with linear decay. The SGD optimizer was used with a momentum of 0.9 and zero weight decay. The classification accuracy was used as the measure of the performance, and all the results reported below were averaged over four runs. We also report the result of CP-ResNet combined with Mixup [45], which is a widely used augmentation method in ASC tasks. For DCASE’20 Low Complexity ASC, the baseline of self-KD methods was frequency damping [44]. A similar training setup was used as for DCASE’18 ASC.

As Table 7 shows, MrKD improved both CP-ResNet and the combination with Mixup by 1.06% and 0.53%, respectively. Although our method improved the baseline performance, no significant improvement was shown comparing to the second-best results of other self-KD methods in Table 7. Note that MSD obtained similar result as our method on CP-ResNet but failed to get improvement when combining with Mixup. We argue that Mixup was a strong augmentation method. In this case, the knowledge offered by standalone peers was no longer helpful, while the similarity of the student and historical model in MrKD made our method effective. Self-KD obtained less improvement on CP-ResNet, both with or without Mixup, than ours. To make this conclusion more concrete, we performed the t-test of the equality of means hypothesis between the results of self-KD and ours. The level of confidence of rejection without and with Mixup was 98.9% and 91.5%, respectively.

The evaluation on DCASE’20 Low Complexity ASC is shown in Table 8. The accuracy drop of baseline with Mixup indicated that as a strong label-mixing augmentation method, Mixup was not effective when the accuracy gap between training set and test set was small. Similar to DCASE’18 ASC, although MSD obtained similar improvement as MrKD on Freq-damp, it failed to improve the combination with Mixup. Self-KD and MrKD obtained improvement consistently on DCASE’20 Low Complexity ASC, while MrKD had higher means. The rejection confidence of the equality of means hypothesis was 97.7% and 98.9%, without and with Mixup, respectively. Particularly, MrKD achieved better performance on DCASE’20 Low Complexity ASC when combined with Mixup augmentation. As illustrated in Figure 1, since our method MrKD redesigned the training procedure and did not modify the final student network, the 500 KB size limit of DCASE’20 Low Complexity ASC was still satisfied.

## 5. Discussion

We investigate MrKD, a self-knowledge distillation method with historical models for improving the DNN classification tasks. Three components are involved in our method: MrKD-plain, FCN ensemble, and Knowledge Adjustment. As the ablation study in Section 4.1 shows, MrKD-plain with only historical model replay boosts the performance most. FCN ensemble can reduce the classification error rate in a certain extent. Knowledge Adjustment makes more obvious progress on deeper networks. The experiment on CIFAR-10 (Table 6) shows that when the error rate gap between training and test set is small, the mixture of backup output and ground truth label in self-KD [40] may perform better than standalone KL loss in MrKD. The difference between self-KD and MrKD is shown in Figure 2.

If one wants to apply the proposed method to other classification problems, it is necessary to modify the FCN network size for the different class amounts. We determine the update frequency κ to be 25 epochs with a simple grid search with the total epoch set as 200. Some problems may need more or less total epoch to train. If the total epoch for training is changed, the update frequency needs to adjust proportionally for optimal performance.

Moreover, one can process the multiple feed-forward propagations of MrKD in parallel. Since the time cost by communication, FCN ensemble, and KA is negligible compared to the forward and backward propagation, the training time will be similar to typical classification solutions with CE loss. However, each extra GPU will cost identical memory as the current student. If only one GPU is available, each model backup will cost an extra 25% of training time for its forward propagation, empirically. In this case, each backup’s extra memory cost is only 2.5% since the GPU only needs to keep one graph structure for all models, and the specific value of each parameter occupies only a small amount of memory. Note that all the analysis above is for the training procedure. For testing or deployment of a trained student network, since the teacher model is abandoned and only the student is used for prediction, the computational cost is identical to a regularly trained model.

The historical models during training trajectory are always considered useless and abandoned immediately after the current model parameters are updated. However, this paper insists on the idea proposed by Self-KD [40]. That is, historical models can also help the current model training by knowledge distillation. Instead of the backup’s output logits, other types of knowledge which have been investigated in traditional KD methods may also be mined from the model backups in future work, such as weights regularization, intermediate layer outputs [46], or attention maps [47,48]. Furthermore, Knowledge Adjustment is proved to be useful in MrKD and may generalize to other online KD methods in which the teacher is prone to make mistakes in the earlier training stage, such as DML [16], CS-KD [19], MSD [21].

## 6. Conclusions

To low-capacity sensor devices, knowledge distillation is an essential technique for model compression. Furthermore, self-knowledge distillation can improve the supervised learning model training directly without the pre-trained teacher in traditional KD methods. In this paper, we propose a simple but effective self-KD method without external knowledge. Adopting model parameter backups as the teachers of self-distillation, MrKD can improve classification problems. Experimental results show that MrKD can decrease the classification error rate of DNN architectures (ResNet, WideResNet, ResNeXt) on image datasets (CIFAR-100, CINIC-10, CIFAR-10) effectively from 0.50% to 1.96%. MrKD also improves the audio classification DCASE’18 ASC and DCASE’20 Low Complexity ASC tasks with 0.3% to 1.0% accuracy raise. The results above indicate that MrKD can improve both image and audio supervised-learning tasks consistently.

The ablation study on CIFAR-100 shows that Fully Connected Network ensembling and Knowledge Adjustment are two useful components for MrKD.

## Figures and Tables

**Table 1:** Summary of different Knowledge Distillation (KD) methods in implementation and computation complexity. Note that we adopt ‘f’ and ‘b’ as units to indicate the forward propagation and backward propagation time of the student network. The results are shown with only one teacher (Born Again Network—BAN and Memory-replay Knowledge Distillation—MrKD), peer (Deep Mutual Learning—DML), or branch (MSD).

KD Method	Need Pre-Trained Teacher	#Parameters Engaged	Need Model Redesign	Training Time	Test Time
BAN [15]	Yes	2×	No	2f + 1b	1f
DML [16]	No	2×	No	2f + 2b	1f
MSD [21]	No	1.75×	Yes	2f + 2b	1f
MrKD	No	1×	No	2f + 1b	1f

**Table 2:** Test error rate comparison on CIFAR-100 [26] dataset (mean (±standard deviation), in %).

Model	#Parameter	Baseline	Self-KD [40]	MSD [21]	MrKD
ResNet-32 [1]	0.5 M	30.08 (±0.57)	28.15 (±0.28)	28.31 (±0.18)	**28.12** (±0.27)
ResNet-56	0.9 M	27.76 (±0.24)	26.24 (±0.35)	**26.18** (±0.27)	26.23 (±0.16)
ResNet-110	1.7 M	25.79 (±0.44)	24.91 (±0.62)	25.38 (±0.35)	**24.76** (±0.15)
ResNet-164	1.7 M	23.46 (±0.31)	21.85 (±0.33)	**21.72** (±0.22)	22.12 (±0.21)
WRN-16-8 [28]	11.0 M	20.35 (±0.37)	19.33 (±0.51)	19.34 (±0.18)	**19.12** (±0.16)
WRN-28-10	36.5 M	19.60 (±0.22)	18.71 (±0.35)	18.56 (±0.16)	**18.32** (±0.09)
ResNeXt-29, 8 × 64d [29]	34.5 M	18.57 (±0.24)	17.55 (±0.31)	17.46 (±0.25)	**17.23** (±0.18)

**Table 3:** Test error rate comparison on CIFAR-100 [26] dataset with different components. The ‘w/o’ stands for ‘without’.

Model	#Parameter	Baseline	MrKD w/o	MrKD w/o	MrKD
			FCN and KA	KA	
ResNet-32 [1]	0.5 M	30.08	28.85	28.17	**28.12**
ResNet-56	0.9 M	27.76	26.74	**26.02**	26.23
ResNet-110	1.7 M	25.79	25.17	24.79	**24.76**
ResNet-164	1.7 M	23.46	22.85	22.46	**22.12**
WRN-16-8 [28]	11.0 M	20.35	19.63	19.24	**19.12**
WRN-28-10	36.5 M	19.60	19.01	18.58	**18.32**

**Table 4:** Test error rate comparison on CIFAR-100 [26] with different loss setting (mean (±standard deviation), in %).

Model	#Parameter	Baseline	CE Loss	KL Loss	CE+KL Loss
ResNet-56	0.9 M	27.96 (±0.42)	26.64 (±0.15)	**26.33** (±0.16)	26.42 (±0.16)
ResNet-110	1.7 M	25.97 (±0.39)	25.21 (±0.23)	**24.76** (±0.15)	24.89 (±0.14)
ResNet-164	1.7 M	23.66 (±0.31)	22.55 (±0.25)	22.12 (±0.21)	**22.02** (±0.16)

**Table 5:** Test error rate comparison on the CINIC-10 [27] dataset (mean (±standard deviation), in %).

Model	#Parameter	Baseline	Self-KD [40]	MSD [21]	MrKD
ResNet20 [1]	0.3 M	17.86 (±0.21)	16.88 (±0.23)	16.75 (±0.09)	**16.62** (±0.08)
ResNet32	0.5 M	16.63 (±0.17)	15.61 (±0.27)	15.76 (±0.08)	**15.55** (±0.14)
ResNet56	0.9 M	15.44 (±0.20)	14.75 (±0.16)	14.91 (±0.07)	**14.74** (±0.07)
WRN-16-8 [28]	11.0 M	11.85 (±0.16)	**11.09** (±0.09)	11.12 (±0.09)	11.15 (±0.06)
ResNeXt-29, 8 × 64d [29]	34.5 M	11.35 (±0.15)	10.25 (±0.22)	10.33 (±0.15)	**10.17** (±0.10)

**Table 6:** Test error rate comparison on the CIFAR-10 [26] dataset (mean (±standard deviation), in %).

Model	#Parameter	Baseline	Self-KD [40]	MSD [21]	MrKD
ResNet20 [1]	0.3 M	7.61 (±0.16)	7.27 (±0.25)	7.14 (±0.15)	**7.08** (±0.15)
ResNet32	0.5 M	6.53 (±0.14)	6.26 (±0.15)	6.18 (±0.17)	**5.96** (±0.15)
ResNet56	0.9 M	6.14 (±0.19)	5.62 (±0.13)	5.73 (±0.09)	**5.58** (±0.21)
WRN-16-8 [28]	11.0 M	4.42 (±0.14)	**3.75** (±0.22)	3.88 (±0.10)	3.81 (±0.11)
ResNeXt-29, 8 × 64d [29]	34.5 M	4.11 (±0.19)	**3.51** (±0.09)	3.66 (±0.08)	3.61 (±0.06)

**Table 7:** Test accuracy comparison on DCASE’18 acoustic scene classification (ASC) [30] dataset.

Model	#Parameter	Baseline	Self-KD [40]	MSD [21]	MrKD
CP-ResNet [43]	3.57 M	77.26	78.08	**78.26**	**78.32**
+Mixup [45]	3.57 M	79.99	80.28	79.03	**80.52**

**Table 8:** Test accuracy comparison on DCASE’20 Low Complexity ASC [31] dataset.

Model	#Parameter	Baseline	Self-KD [40]	MSD [21]	MrKD
Freq-damp [44]	0.25 M	97.05	97.22	**97.32**	**97.34**
+Mixup [45]	0.25 M	96.65	97.37	96.84	**97.52**

## References

[1] He K., Zhang X., Ren S., Sun J. Deep residual learning for image recognition. Proceedings of the IEEE Conference on Computer Vision and Pattern Recognition.

[2] Huang G., Liu Z., Pleiss G., Van Der Maaten L., Weinberger K. (2019). Convolutional Networks with Dense Connectivity. IEEE Trans. Pattern Anal. Mach. Intell..

[3] Chen Y., Li J., Xiao H., Jin X., Yan S., Feng J. (2017). Dual path networks. Adv. Neural Inf. Process. Syst..

[4] Howard A.G., Zhu M., Chen B., Kalenichenko D., Wang W., Weyand T., Andreetto M., Adam H. (2017). MobileNets: Efficient Convolutional Neural Networks for Mobile Vision Applications. arXiv.

[5] Sandler M., Howard A., Zhu M., Zhmoginov A., Chen L.C. Mobilenetv2: Inverted residuals and linear bottlenecks. Proceedings of the IEEE Conference on Computer Vision and Pattern Recognition.

[6] Howard A., Sandler M., Chu G., Chen L.C., Chen B., Tan M., Wang W., Zhu Y., Pang R., Vasudevan V. Searching for MobileNetV3. Proceedings of the 2019 IEEE/CVF International Conference on Computer Vision (ICCV).

[7] Ma N., Zhang X., Zheng H.T., Sun J. Shufflenet v2: Practical guidelines for efficient cnn architecture design. Proceedings of the European Conference on Computer Vision (ECCV).

[8] Liu H., Simonyan K., Yang Y. (2018). DARTS:Differentiable Architecture Search. arXiv.

[9] Tan M., Le Q., Chaudhuri K., Salakhutdinov R. EfficientNet: Rethinking Model Scaling for Convolutional Neural Networks. Proceedings of the 36th International Conference on Machine Learning.

[10] Hinton G., Vinyals O., Dean J. (2015). Distilling the Knowledge in a Neural Network. arXiv.

[11] Cho J., Lee M. (2019). Building a Compact Convolutional Neural Network for Embedded Intelligent Sensor Systems Using Group Sparsity and Knowledge Distillation. Sensors.

[12] Park S., Heo Y.S. (2020). Knowledge Distillation for Semantic Segmentation Using Channel and Spatial Correlations and Adaptive Cross Entropy. Sensors.

[13] Choi E., Chae S., Kim J. (2019). Machine Learning-Based Fast Banknote Serial Number Recognition Using Knowledge Distillation and Bayesian Optimization. Sensors.

[14] Chechlinski L., Siemiątkowska B., Majewski M. (2019). A System for Weeds and Crops Identification—Reaching over 10 FPS on Raspberry Pi with the Usage of MobileNets, DenseNet and Custom Modifications. Sensors.

[15] Furlanello T., Lipton Z.C., Tschannen M., Itti L., Anandkumar A. Born Again Neural Networks. Proceedings of the International Conference on Machine Learning.

[16] Zhang Y., Xiang T., Hospedales T.M., Lu H. Deep Mutual Learning. Proceedings of the IEEE Conference on Computer Vision and Pattern Recognition (CVPR).

[17] Gao L., Lan X., Mi H., Feng D., Xu K., Peng Y. (2019). Multistructure-Based Collaborative Online Distillation. Entropy.

[18] Zhang L., Song J., Gao A., Chen J., Bao C., Ma K. Be Your Own Teacher: Improve the Performance of Convolutional Neural Networks via Self Distillation. Proceedings of the IEEE/CVF International Conference on Computer Vision (ICCV).

[19] Yun S., Park J., Lee K., Shin J. Regularizing Class-Wise Predictions via Self-Knowledge Distillation. Proceedings of the IEEE/CVF Conference on Computer Vision and Pattern Recognition (CVPR).

[20] Xu T.B., Liu C.L. (2019). Data-Distortion Guided Self-Distillation for Deep Neural Networks. Proc. AAAI Conf. Artif. Intell..

[21] Luan Y., Zhao H., Yang Z., Dai Y. (2019). MSD: Multi-Self-Distillation Learning via Multi-classifiers within Deep Neural Networks. arXiv.

[22] Hendrycks D., Mu N., Cubuk E.D., Zoph B., Gilmer J., Lakshminarayanan B. (2019). AugMix: A Simple Data Processing Method to Improve Robustness and Uncertainty. arXiv.

[23] Mnih V., Kavukcuoglu K., Silver D., Rusu A.A., Veness J., Bellemare M.G., Graves A., Riedmiller M., Fidjeland A.K., Ostrovski G. (2015). Human-level control through deep reinforcement learning. Nature.

[24] Mandt S., Hoffman M.D., Blei D.M. (2017). Stochastic Gradient Descent as Approximate Bayesian Inference. arXiv.

[25] Wen T., Lai S., Qian X. (2019). Preparing Lessons: Improve Knowledge Distillation with Better Supervision. arXiv.

[26] Krizhevsky A., Hinton G. (2009). Learning Multiple Layers of Features from Tiny Images.

[27] Darlow L.N., Crowley E.J., Antoniou A., Storkey A.J. (2018). CINIC-10 is not ImageNet or CIFAR-10. arXiv.

[28] Zagoruyko S., Komodakis N. (2016). Wide Residual Networks. arXiv.

[29] Xie S., Girshick R.B., Dollár P., Tu Z., He K. Aggregated Residual Transformations for Deep Neural Networks. Proceedings of the 2017 IEEE Conference on Computer Vision and Pattern Recognition (CVPR).

[30] Mesaros A., Heittola T., Virtanen T. A multi-device dataset for urban acoustic scene classification. Proceedings of the Detection and Classification of Acoustic Scenes and Events 2018 Workshop (DCASE2018).

[31] Heittola T., Mesaros A., Virtanen T. Acoustic scene classification in DCASE 2020 Challenge: Generalization across devices and low complexity solutions. Proceedings of the Detection and Classification of Acoustic Scenes and Events 2020 Workshop (DCASE2020).

[32] Song G., Chai W. (2018). Collaborative learning for deep neural networks. arXiv.

[33] Lan X., Zhu X., Gong S. (2018). Knowledge distillation by on-the-fly native ensemble. arXiv.

[34] Cho J.H., Hariharan B. (2019). On the Efficacy of Knowledge Distillation. arXiv.

[35] Mirzadeh S.I., Farajtabar M., Li A., Levine N., Matsukawa A., Ghasemzadeh H. (2019). Improved Knowledge Distillation via Teacher Assistant. arXiv.

[36] Jin X., Peng B., Wu Y., Liu Y., Liu J., Liang D., Yan J., Hu X. Knowledge Distillation via Route Constrained Optimization. Proceedings of the IEEE/CVF International Conference on Computer Vision (ICCV).

[37] Izmailov P., Podoprikhin D., Garipov T., Vetrov D., Wilson A.G. (2018). Averaging Weights Leads to Wider Optima and Better Generalization. arXiv.

[38] Tarvainen A., Valpola H., Guyon I., Luxburg U.V., Bengio S., Wallach H., Fergus R., Vishwanathan S., Garnett R. (2017). Mean teachers are better role models: Weight-averaged consistency targets improve semi-supervised deep learning results. Advances in Neural Information Processing Systems 30.

[39] Xu Y., Xu Y., Qian Q., Li H., Jin R. (2020). Towards Understanding Label Smoothing. arXiv.

[40] Kim K., Ji B., Yoon D.Y., Hwang S. (2020). Self-Knowledge Distillation: A Simple Way for Better Generalization. arXiv.

[41] Chen D., Mei J.P., Wang C., Feng Y., Chen C. (2020). Online Knowledge Distillation with Diverse Peers. Proc. AAAI Conf. Artif. Intell..

[42] Wu G., Gong S. (2020). Peer Collaborative Learning for Online Knowledge Distillation. arXiv.

[43] Koutini K., Eghbal-zadeh H., Dorfer M., Widmer G. The Receptive Field as a Regularizer in Deep Convolutional Neural Networks for Acoustic Scene Classification. Proceedings of the European Signal Processing Conference (EUSIPCO).

[44] Koutini K., Henkel F., Eghbal-Zadeh H., Widmer G. Low-Complexity Models for Acoustic Scene Classification Based on Receptive Field Regularization and Frequency Damping. Proceedings of the Detection and Classification of Acoustic Scenes and Events 2020 Workshop (DCASE2020).

[45] Zhang H., Cisse M., Dauphin Y.N., Lopez-Paz D. (2018). mixup: Beyond Empirical Risk Minimization. arXiv.

[46] Romero A., Ballas N., Ebrahimi Kahou S., Chassang A., Gatta C., Bengio Y. (2014). FitNets: Hints for Thin Deep Nets. arXiv.

[47] Zagoruyko S., Komodakis N. (2017). Paying More Attention to Attention: Improving the Performance of Convolutional Neural Networks via Attention Transfer. arXiv.

[48] Aguilar G., Ling Y., Zhang Y., Yao B., Fan X., Guo E. (2020). Knowledge Distillation from Internal Representations. Proc. AAAI Conf. Artif. Intell..

